# Machine learning and the quest for objectivity in climate model parameterization

**DOI:** 10.1007/s10584-023-03532-1

**Published:** 2023-07-18

**Authors:** Julie Jebeile, Vincent Lam, Mason Majszak, Tim Räz

**Affiliations:** 1 Institute of Philosophy, University of Bern, Länggassstrasse 49a, 3012 Bern, Switzerland; 2 Oeschger Center for Climate Change Research, University of Bern, Hochschulstrasse 6, 3012 Bern, Switzerland; 3 CNRM UMR 3589, Météo-France/CNRS, Centre National de Recherches Météorologiques, Toulouse, France; 4 The University of Queensland, School of Historical and Philosophical Inquiry, 4072 St Lucia QLD, Australia

**Keywords:** Climate modeling, Parameterizations, Parameter tuning, Objectivity, Subjectivity, Expert judgement, Machine learning, Deep neural networks, Gaussian processes

## Abstract

Parameterization and parameter tuning are central aspects of climate modeling, and there is widespread consensus that these procedures involve certain subjective elements. Even if the use of these subjective elements is not necessarily epistemically problematic, there is an intuitive appeal for replacing them with more objective (automated) methods, such as machine learning. Relying on several case studies, we argue that, while machine learning techniques may help to improve climate model parameterization in several ways, they still require expert judgment that involves subjective elements not so different from the ones arising in standard parameterization and tuning. The use of machine learning in parameterizations is an art as well as a science and requires careful supervision.

## Introduction

Machine learning applications in science are receiving more philosophical attention as they raise significant epistemic issues, related to interpretability and understanding (see Beisbart and Räz 2022; Räz and Beisbart 2022 as well as references therein). Some discussions have focused on the climate context more specifically (e.g. Jebeile et al. 2021; Knüsel and Baumberger 2020; Kawamleh 2021), as there is high expectation from the scientific community that machine learning can reduce computational cost and overcome climate model uncertainty. Another important expectation however has been overlooked by philosophers: the hope that machine learning can alleviate the subjectivity of some parts of climate models, in particular the parameterizations and their associated parameter tuning. In this paper, we aim to explicate the sources of subjectivity in climate model parameterization and tuning, and subsequently investigate whether, and how, the diverse available machine learning techniques can make climate models more objective.

Models can represent the climate system only up to a certain spatial resolution (grid size), due to numerical constraints and the computational cost of higher resolutions.^1^ The physical laws, e.g., the Navier–Stokes equations, on which models rely, are thus discretized on a grid to be implemented on the computer and numerically solved in manageable timeframes. Therefore, processes such as convection (clouds), which typically occur at the sub-grid level and are relevant to climate modeling, need to be represented using so-called parameterizations. Parameterizations are simplified representations—also qualified as “mini-models” (Lloyd 2015,61)—of these processes based on phenomenological and theoretical considerations (see also Guillemot 2017 and Winsberg 2018, 47-50). The phenomenological considerations involve many parameters (hence the term ‘parameterization’), some of which are just consequences of the discretization and parameterization procedures and thus do not correspond to anything in the target system. Parameter tuning is intrinsically part of the building of parameterizations, it is the crucial (and complex) process of adjusting the parameters to a predefined set of observations (and for a given purpose).

Because the design of parameterizations and parameter tuning are made under high uncertainty and are complex, they face underdetermination and leave leeway for subjective decisions. Parameterizations have been identified as one of the main sources of uncertainty in climate models and come with both structural uncertainty and parameter uncertainty, as there is no unique way to parameterize sub-grid processes. Given a certain parameterization procedure, there is not necessarily any ‘true’ value for the involved parameters, but rather some (not necessarily unique) ‘best’ values that are adequate for given purposes (see, e.g., the discussion in Winsberg 2018, 47-49); the choice of parameter values in parameterizations is usually not sufficiently constrained by theory or observations. It has been highlighted that parameterizations may be merely heuristic as opposed to being physical (e.g. Rasp et al. 2018), and that parameter tuning is carried out by experts “by hand” and in an ad hoc manner, involving a certain degree of subjectivity (see e.g. Hourdin et al. 2017).

Recently, climate modelers have proposed to overcome this unsatisfactory situation with the help of machine learning (ML) models. Not only could ML models improve parameterizations by reducing computational cost but may also help with uncertainty quantification (e.g. Rio et al. 2019). Some climate modelers have also suggested that utilizing ML models can reduce the subjectivity involved in parameterization and parameter tuning. Intuitively, it seems uncontroversial that, *ceteris paribus*, objective methods are epistemically more valued than subjective ones—leading to what we call ‘the quest for objectivity’.

We will critically assess the notion that utilizing ML models will make parameterizations strictly more objective. We begin by exemplifying how traditional parameterizations and parameter tuning are performed in practice, using the canonical example of cloud parameterization (Sect. 2.1). In particular, we identify three interrelated subjective elements at play in the use of expert judgment in parameterizations (Sect. 2.2). We then introduce a distinction between different camps of climate modelers, depending on how far they are willing to push the use of ML models to improve parameterizations, and examine the ML methods to be used by the different camps (Sect. 3). What we call the “conservatives” (e.g. Schneider et al. 2017b; Couvreux et al. 2020) want to retain traditional parameterizations, but propose to automatize parameter tuning with ML models such as Gaussian processes (Sect. 3.2). What we call the “progressives” (e.g. Gentine et al. 2018; Rasp et al. 2018) propose to push the use of ML models further by replacing entire parameterization modules with ML models, e.g., deep neural networks (DNNs) (Sect. 3.4). In both cases, we argue that the use of (different) ML models introduces the need for (different kinds of) expertise. We maintain that the implementation of ‘objective’ and ‘automated’ machine learning techniques involves subjective elements that are very similar to the ones arising in standard parameterization and tuning procedures—in this sense, machine learning does not make much progress in the quest for objectivity (which is not necessarily problematic from the epistemic point of view) (Sect. 4). We believe that ML models may help improve parameterizations in several ways, but they still require expert choices and should not be considered to be objective alternatives to traditional methods.

## Traditional parameterizations

### Overview

This section will illustrate how traditional parameterizations are usually built, with specific attention given to the role of experts and their subjective imprint on the design of models through expert judgment; this case will then be compared with machine learning techniques.^2^

In climate models, there is a variety of parameterizations for representing microphysics, radiation, turbulence and convection. For the sake of illustration, let us take the example of convective parameterization. It provides crucial input to the dynamic and radiation equations in General Circulation Models or GCMs (Rio et al. 2019), yet the various current versions of convective (and cloud) parameterization are responsible for the most significant part of model spread (Stevens and Bony 2013) and their development is suspected to have reached a “cloud parameterization deadlock” (Randall et al. 2003). Atmospheric convection and its associated clouds operate at scales (around 1 km) that cannot be described explicitly in GCMs, which currently have a coarse spatial resolution (around 10-100 km). Thus, convective parameterization is used to account for the collective effect of cumulus clouds at the scale of the model grid. Like any other parameterization, it is a “semi-empirical” component (Edwards 2010) and turns climate models into “hybrid models” (Katzav 2013), as it is neither purely empirical (phenomenological) nor entirely theory-based. Historically, in the 1960s, convective parameterization was mainly phenomenological as it contained *ad hoc* hypotheses, i.e., moist convective adjustments based on observations, whose goal was to overcome numerical instabilities (Touzé-Peiffer 2021). Later on, in the 1980s and 1990s, the majority of the convective parameterizations were based on the equations of fluid dynamics and differ in their way to decompose the atmospheric components and design the different levels of convective organizations. The mass-flux approach, which is still predominant, represents explicitly the convective physical processes, based on the decomposition of the atmosphere into updrafts, downdrafts, and boundary layer. More recent developments focus on utilizing stochastic components, the development of new prognostic equations or the inclusion of new processes such as cold pools and mesoscale organization (between shallow and deep convective cells) (see Rio et al. 2019).

When parameterizations take mainly the form of phenomenological laws, expert judgment is used to infer approximate yet relevant relationships between variables of interest based on the available observations. Thus, these parameterizations require a high degree of inductive skills from the modelers. When parameterizations are theory-based laws, they encode, to some extent, the physics of atmospheric convection and cloud formation. Here “parameterizations summarize our understanding of physical processes and their interactions with the large-scale flow” (Rio et al. 2019, 96). A certain number of omissions and distortions (‘idealizations’) are made though, as parameterizations are necessarily approximate representations. Therefore, in this case, expert judgment is used to choose those simplifying assumptions based, for instance, on a trade-off between representational accuracy of the parameterization, i.e. its capacity to describe properly the physical processes, on the one hand, and computational cost of the parameterization, i.e. the computational time to perform the calculation on the other hand. To the extent that such expert judgment is not (fully) determined by observational data or theory, interests and values may also influence the way parameterizations are shaped for the models to meet specific purposes. One example is the preference of representing climate variables and phenomena that are prioritized by the modelers, e.g., variables and phenomena relevant for the regions they live in.

Parameterization goes hand in hand with parameter tuning as the parameters in parameterizations need to be adjusted. Thus, a distinction is made between uncertainty in the formulation of parameterization schemes, which is linked to structural uncertainty, and uncertainty in the choice of parameter values, i.e. parameter uncertainty. Parameter tuning is actually a mix of very different methods, which can be broadly categorized as ‘objective’ and ‘subjective’. By objective methods, Hourdin et al. (2017, 594) mean “that a well-founded mathematical or statistical framework is used to perform the model tuning, for instance, by defining and minimizing a cost function or by introducing a Bayesian formulation of the calibration problem”. Accordingly, tuning can be seen as an optimization problem, minimizing a function that evaluates the distance between some model outputs and selected observations.^3^ On the other hand, several crucial stages of the tuning process require subjective judgment in the concrete sense of choices that are made by the modelers and that are not determined by observational data or theory. First, the tuning process, when made manually, is sometimes characterized as ‘artisanal’: “The model tuning process at our institute is artisanal in character, in that both the adjustment of parameters at each tuning iteration and the evaluation of the resulting candidate models are done by hand, as is done at most other modeling centers.” (Mauritsen et al. 2012, 16). Second, decisions regarding the target variables to tune and the optimization metrics are also said to involve knowledge and intuition of the modelers (Mauritsen et al. 2012; Hourdin et al. 2017; Schmidt et al. 2017), and more particularly knowledge and intuition “about plausible ranges of the tunable parameters and about the effect of parameter changes on the simulated climate of a model" (Schneider et al. 2017b, 12’397). Third, in parameter tuning, decisions can depend on the value-driven purposes and priorities of modelers (see Intemann 2015). For the design of parameterization schemes, they might depend on the geographical origin of the modelers. Thus, a modeling group may have to choose between optimizing ocean heat transport in the North Atlantic or tropical convection (Hourdin et al. 2017, 592). Any choice between the two may have unforeseeable consequences on other model outputs down the road, because of the holism and epistemic opacity at work in complex climate models such as the state-of-the-art GCMs (Lenhard and Winsberg 2010).

### Subjective elements

From this description, we see three different, yet interrelated, types of subjectivity at play in the use of expert judgment in parameterizations. The evaluation of expertise generally focuses on the social connection between the layperson and the expert, as expert judgment is seen as an instance of testimonial knowledge.^4^ However, we are focused on a different epistemic situation, where there is not a layperson eliciting a judgment from an expert, rather there is a single expert, or group of experts, using their own judgment(s) to build a parameterization and subsequently the entire model. Therefore, in this paper, we focus on three interrelated expert traits, namely the expert’s content-knowledge, the expert’s use of values/biases and the expert’s practical experience or track record.^5^

#### Inductive skill / content knowledge

The design of a phenomenological law is a nontrivial procedure that utilizes the inductive skills of the modelers for the identification of the relevant variables and the elaboration of the empirical relationships between those variables. While some argue, in line with Hume’s skeptical viewpoint, that inductions are epistemically problematic, this form of reasoning, at the very least, has a strong history of use in science with varying levels of empirical success (Douven forthcoming).

The specific type of induction used in the building of parameterizations is done by using the expert’s content-knowledge. This is the knowledge experts gain about the domain of interest, from their education and understanding (Martini 2020), this can be exemplified as theoretical considerations, observational and numerical data, current state-of-the-art research, ongoing deliberations and debates, as well as other factors. In this case, experts take what they know about the target, that aspect of the climate which is aimed to be represented, and infer a representational relationship between the real world target to some identified phenomenological law. The judgment in question is then in the identification of both the phenomenological law and the representational relationship, and the expert must ask themselves: given a certain purpose, to what extent does the identified phenomenological law adequately capture the relevant aspects of the target within the parameterization? From this, the subjectivity and subsequent epistemic justification for the specific expert judgment are contingent on two aspects of the expert, their inductive reasoning skills and their set of knowledge to reason from, which can involve some (e.g. methodological) preferences when ambiguities are present.

This combination of content knowledge and inductive skill can also be seen at work during the tuning process. As previously mentioned, the decision making regarding the plausible ranges for the identified tunable parameters requires content knowledge and inductive skill. For a given tunable parameter, the experts must identify the relevant range of values, given their knowledge of the system and of the given parameter which is being tuned. The experts must then use their inductive skills to foresee how these newly tuned parameters will further affect the model and judge if the representational relationship between model and target still holds based on the purpose of the model. Thus, the judgment is qualified by the content-knowledge available to the expert at the time of the judgment and the experts ability to infer from this set of knowledge. In turn, the justification for the specific expert judgment must then stem from the abilities and features of this specific expert.

#### Practical experience / track record

In the choice of idealizations, the kinds of omissions and distortions previously discussed illustrate the subjective decision making at work in choices between representational accuracy and computational cost of the parameterization. The decision making is also guided by the practical experience of the modeler on how to concretely build the parameterization. One can characterize this kind of expertise as practice based, through time a modeler gains expertise by working with their model, developing a kind of tacit knowledge or a kind of know-how. Generally, this type of expertise is used in contexts like riding a bike, where you can learn or know all the mechanical processes of how to ride a bike, but until you get on a bike and start peddling for yourself, you will not acquire this know-how. While interrelated, this practical experience can be contrasted with the more theoretical experience discussed in the context of content knowledge.

The expert decision is then strongly rooted in the experiences the expert has had, knowing how to perform a task to reach the desired goal, like in this case building a model parameterization. Thus, the type of subjectivity found in these judgments is relatively unproblematic from an epistemic point of view, given that certain qualities of the expert are met. The expert must have the requisite track record or history of working with parameterizations and climate models. We take track record to be an account of the expert’s credentials, their publishing record and academic background, and relevant experience in relation to the judgment that has been elicited (Martini 2020). In brief, if the expert has a history of working with a specific model and developing parameterizations that represent the specific phenomena in question, then it can be said that they have a track record and the required know-how for making the decision. In light of this, the degree of epistemic worry then falls on how robust the track record of the expert is, ultimately providing the justification, or lack thereof, for the decisions made in the choice of idealizations. This results in a gradient of epistemic justification for the idealizations, as there can be situations where new parameterizations are developed that are slightly outside the experience of the expert, giving these judgments slightly less justification, or situations of the other variety where the expert may have a robust track record and strong justification for their judgments.

#### Values / biases

The use of (non-epistemic) values in building parameterizations and in the identification of which parameters to tune, can be seen as focusing the inquiry, as in targeting a specific type of phenomena (or variable) or geographic region for greater representational accuracy within the model. From this, the expert trait of unbiasedness comes to the forefront (Martini 2020), where these subjective biases can be social, political or even cognitive elements of the expert. It is argued that the quality or trustworthiness of an expert judgment should be, in part, dependent on if there are clear biases instituted by the expert, meaning if biases are found, then the judgment is unjustified and should not be used. With this conception of unbiasedness it is clear that the use of values to focus the domain of inquiry, as described above, does show biases in the expert judgments, as preferring to represent variables or tune parameters which are relevant for a certain region rather than another is purely subjective and institutes clear biases. However, we argue that this should not be the end of the discussion, rather we should investigate if the biases are epistemically problematic.

We foresee two potential epistemic problems, one of epistemic access and the other relating to the quality or justification of information provided by the model. The use of values as described does limit the scope of a given model, and thus the epistemic access it can provide, as a model will not be able to equally represent every phenomenon and geographic region. Some may argue that this is not overly problematic, as we are referencing the construction of a single parameterization within a specific model and not every model. It would be epistemically problematic however if all modeling efforts used the same set of values, this would result, for instance, in the modeling community being unable to gain accurate information about some specific phenomena or areas of the world. Unfortunately this seems to be the case to a certain extent, as every region of the world does not have an equal voice in the scientific community for value selection—typically, the modeling community is rather dominated by the Global North.

The second problem relates to the quality of the information that can be elicited from a model. With some aspects of the model having greater representational accuracy, due to the scope of the model, there is a greater epistemic justification for that information produced within the scope or purpose of the model. In turn, information produced about phenomena or geographic locations that fall outside the scope will not be as epistemically justified, as the adequacy for purpose can be brought into question. This obviously concerns the tuning process as well, where, as we have seen, not all relevant aspects (variables) of the target may be optimized within the model. However, in so far as those models are used in line with their intended purpose, then this practice is once again not too epistemically problematic (Parker 2020). What might make this problem more tricky to disentangle would be if different values were used across different aspects of the modeling effort, as if multiple and conflicting values were used across the different parameterizations and tuning methods of a single model. If this situation were to occur this could bring the epistemic justification or the reliability of the model outputs into question as there would not be a clear purpose or epistemic scope. Concretely, it would be extremely difficult to identify (and remedy) such situations, since modeling efforts are compounded on previous efforts, resulting in the purpose of some lines of code not being explicitly known to the modelers building the subsequent features of the model (Lenhard and Winsberg 2010). What remains clear is that the use of values should be as internally consistent as possible, so as to identify the correct scope of information that can be epistemically justifiable from a given model. Thus, as it currently stands, this subjective element continues to play an important role in the design of parameterizations, for framing how the model can and should be used, but their use must be evaluated on a case by case basis.

There is a general intuitive appeal for eliminating, or at least minimizing, these subjective elements, that is, for replacing them with more objective methods. This is suggested in Mauritsen et al. (2012, 16), when they write—right after characterizing the tuning process as being “artisanal”—that it is “at least conceptually possible to automate this process and find optimal sets of parameters with respect to certain targets”. Of course, there are in principle different possible ways to achieve this, but machine learning methods as automatic processes are very natural candidates because, as we will see in what follows, these methods provide an avenue to, in theory, remove some subjective elements.^6^ Indeed, in recent years, machine learning techniques have been applied to climate model parameterization, and in particular to atmospheric convection parameterization (see for instance Schneider et al. 2017b, Gentine et al. 2018, O’Gorman and Dwyer 2018, Rasp et al. 2018, Couvreux et al. 2020).^7^

Before turning to the case studies, it is important to note that the application of machine learning to cloud parameterization runs in parallel, and is in some sense complementary, to the development of cloud-resolving models (CRMs), which are high resolution models with kilometric horizontal resolution that explicitly represent relevant cloud-related physical processes.^8^ While resolving convection would alleviate some of the above mentioned subjectivity, it is extremely computationally costly and thus not implementable at a large scale in the foreseeable future. So-called super-parameterizations (SP), which involve (simplified) CRMs embedded in the grid cells of a GCM, constitute an intermediate strategy. In this context, CRM outputs can be used to train machine learning algorithms, which are expected to reduce the very high computational costs of cloud-resolving models—and to further reduce the subjective elements linked to parameterization.

## Machine learning and parameterization: case studies

### Overview

In this section, we provide detailed case studies to concretely show this push for more objectivity through ML models. We examine cases in which ML models are used in different ways to overcome “subjective elements”, or “heuristic arguments” in traditional parameterizations. ML can be used for different purposes at different levels in Global Circulation Models (GCMs) or Earth System Models (ESMs) (see Reichstein et al. 2019), and, depending on the purpose, different kinds of ML are appropriate.

We distinguish, somewhat programmatically, between two ways of improving parameterizations using ML, depending on how much of traditional parameterizations the respective proposals are willing to sacrifice. First, the *conservatives* want to retain existing parameterizations, because of their built-in physicality and other advantages, but advocate utilizing ML methods in parameter tuning. Second, the *progressives* propose to replace entire parameterizations with ML models, such as DNNs.

We will show that in both camps, some claim that the use of ML methods removes subjective elements and leads to more objectivity in the parameterization context. However, the two camps use different ML methods to achieve their respective goals, and these methods vary in the degree to which they lead to ‘objectivity’. The diverging degrees to which the two camps are willing to abandon traditional parameterization may be rooted in different attitudes towards the reliability or ad-hoc-ness of the parameterization schemes themselves. Progressives, who consider parameterizations to be somewhat ad-hoc, may be more willing to replace the parameterizations with ML methods that come with a certain inductive risk and may not be very well understood. Conservatives, who consider parameterizations to be (at least partly) well-founded in physical principles, may be less willing to remove them. This difference may ultimately be tied to different (subjective) attitudes towards the structural uncertainty associated with parameterizations.

### The conservatives

Schneider et al. (2017b) propose to retain parameterization schemes that incorporate physical, chemical and biological knowledge, but to tune parameters in a novel way, based on global observations and high-resolution simulations. Schneider et al. illustrate their proposal with the Lorentz-96 model, a simple dynamical model. They generate data from this model and use two ML methods, the so-called Bayesian inversion approach (MCMC), as well as the Kalman ensemble approach, to estimate four parameters of this model. This proof-of-concept is encouraging, but also limited in scope: by using simulation data, it is not possible to gauge what happens if there is not only parameter uncertainty, but also structural uncertainty.

Schneider et al. argue against discarding parameterizations that are based on theory: "The machine learning of parameterizations in our view should be informed by the governing equations of subgrid-scale processes whenever they are known" (p. 12,409). By doing so, one avoids the problem of "unstructured parameterization schemes", e.g., DNNs, which do not satisfy symmetries and conservation laws. Unstructured parameterization schemes may lead to poor out-of-sample performance, which is very undesirable in the context of climate change scenarios. This is the main argument against the progressive camp. However, Schneider et al. also argue that their approach is more flexible than the "traditional approach of fixing closure parameters ad hoc or on the basis of a small sample of observations or high-resolution simulations" (p. 12,410). They note that "for non-computable processes whose governing equations are unknown, like many ecological or biogeochemical processes, more empirical, data-driven parameterization approaches may well be called for" (p. 12,410). This argument against traditional parameterization schemes can be interpreted as a push for more objectivity, in that the “ad hoc-ness” of traditional parameter tuning is avoided.

Schneider et al. acknowledge that utilizing ML methods brings about a different set of challenges and requires expert knowledge in its own right. The first challenge is that in order to use a ML model in parameter tuning, one has to choose an appropriate objective function (also known as loss function), which encodes the optimization problem to be solved by the ML models. The objective function should incorporate both bias correction (correction of empirically observed systematic errors in ESMs) and so-called emergent constraints (empirically observed high-level relationships between observable quantities). Schneider et al. write that determining an appropriate objective function requires domain expertise: "In practice, the choice of objective functions will be guided by expertise specific to the relevant subdomains of Earth system science, as well as computational cost" (p. 12,401). The second challenge is that utilizing ML models also necessitates the choice of a learning algorithm that carries out the optimization. Schneider et al. discuss three possibilities: ordinary least squares regularization, Markov Chain Monte Carlo (MCMC) methods, and ensemble methods (ensemble Kalman methods). The ultimate choice of ML (or statistical) method at this point involves making a tradeoff “between computational expense and the amount of information about the parameters they provide” (p. 12,403). This tradeoff, in turn, is ultimately in the hand of experts.

Couvreux et al. (2020) take a similar approach as Schneider et al. (2017b), in that they too aim to improve parameter tuning. They use ML models to emulate a traditional parameterization, a so-called single column model (SCM). The idea is to use the point predictions from SCM runs as training data for the emulator, a so-called Gaussian Process (GP) model. The predictions from the SCM emulator (GP) can then be compared to those of the so-called reference (ground truth), the predictions of a high-resolution simulation (LES ensemble). The comparison uses so-called history matching. The comparison with history matching can lead to a rejection of parts of the parameter space as implausible.

The choice of this approach is justified with two arguments. On the one hand, there is an argument for retaining parameterizations: "[The choice of the strategy] is motivated by the fact that parameterizations summarize our current understanding of the dynamics and physics of atmospheric processes and offer the power of interpretation, crucial to build our confidence in the extrapolation beyond observed conditions realized by any climate projections" (Ibid., p. 3) This is an argument against the progressive camp. On the other hand, it is desirable to systematize the use of parameterizations: "In the proposed approach, machine learning is harnessed in a principled way to calibrate parameterizations at process level. [...] Such a systematic use is not feasible however without more objective and automatic methods than the traditional trial/error approach used to fix parameter values during the parameterization development" (p. 3). Thus, utilizing ML is supposed to make the tuning process more systematic and objective, in contrast to a more ad-hoc trial and error approach, which is ultimately subjective. Further important reasons for their approach, mentioned in the conclusion, are computational speed-up and to explicate different kinds of uncertainty.

A third example from the conservative camp is McNeall et al. (2020), who use Gaussian processes for bias correction in parameter tuning. They acknowledge that “[s]etting input parameters traditionally relies heavily on insights from those involved in parameterisation”, and continue: “this can be an imperfect process, leaving open questions about whether any subsequent simulated biases result from mis-set parameters or wider structural model errors” (p. 2488). McNeall et al. contrast subjective with algorithmic approaches: “Although climate model tuning is overall a subjective process, individual parts of the process are amenable to more algorithmic approaches” (p. 2489). However, they also write that the main goal of using GPs is to help modelers identifying those parts of a model that “would most benefit from development” (p. 2506). Thus, for McNeall et al., the goal of using ML models is not objectivity or automation, but to improve climate models themselves.

Not all representatives of the conservative camp mention the objectivity of ML models as a motivation to use them for parameter tuning. Proske et al. (2021) use Gaussian processes to conduct a sensitivity analysis of parameterizations of cloud microphysical processes. They do not wish to replace entire parameterizations with ML models, and follow Couvreux et al. (2020) in using GP to build more interpretable climate models. They explicitly refer to Rudin (2019), a machine learning scholar who emphasizes the need for physical and interpretable models, as opposed to black-box models. The motivation of Proske et al. is not to make the parameter tuning more objective, but to use it as a tool to gain understanding of the working of climate models themselves.

### Gaussian processes (ML for conservatives)

We have now seen how ML models are used in parameter tuning, and that their use is at least sometimes motivated by the quest for objectivity. In this section, we examine the ML model that is most often mentioned by the conservative camp, Gaussian processes (GPs), and consider to what extent utilizing GPs itself is an objective and automated matter, and whether it involves expertise.

Consider how GPs are used in tuning parameterizations. The use of GPs in combination with history matching to automatize parameter tuning, as proposed by Couvreux et al. (2020) has previously been used by Williamson et al. (2013). The GP is fit to the training data using an adaptive procedure with two stages (Ibid., Appendix B). While this procedure is somewhat automatic, it also involves choices at critical points. For example, different kinds of basis functions can be chosen for the adaptive procedure, depending on the nature of the problem. Williamson et al. describe the transition to the second stage of the adaptive procedure as follows: “When it becomes clear that adding more terms is not improving the predictive power of the emulator (a judgement made by the statistician based on looking at the proportion of variability explained by the regression and at plots of the residuals from the fit) we begin a backwards elimination algorithm" (p. 1725), meaning that utilizing this ML procedure involves a different, statistical, kind of expertise.

A standard textbook on GPs (Williams and Rasmussen 2006) provides a more theoretical perspective. The authors note that one important problem of the application of GPs is the model selection problem, which deals with the question of how to choose the right form of GPs (covariance functions, hyperparameters, parameters). Williams and Rasmussen write: “[...] model selection is essentially open ended. Even for the squared exponential covariance function, there is a huge variety of possible distance measures. However, this should not be a cause for despair, rather seen as a possibility to learn” (Ibid., Sec 5.1.). This means that while it is possible to provide guidance for model selection and make informed choices, the process is open ended rather than automated.

Finally, note that expert judgment is required for history matching, the framework in which GPs are used for parameter tuning.^9^ As noted above, the goal of history matching is to rule out regions of the parameter space as implausible. This is done via an implausibility measure, a distance between reference and emulator prediction (see, e.g., Couvreux et al. 2020, equation (5)). Rejecting parts of the parameter space on the basis of the implausibility measure involves a judgment about structural model error, also known as model discrepancy, because high values of the implausibility measure can be due to an inherent inability of the traditional parameterization (here: SCM) to reproduce the reference. In a paper applying history matching to an ocean model, this judgment is described as follows: “Since ‘structural error’ is ‘real’, for any given metric, we might think of this as a random quantity that could be estimated using a combination of expert modeller judgement and information from dynamic observations and process-based high-resolution simulations" (p. 1794 Williamson et al. 2017). The idea, as Williamson et al. detail, is that the expert has to use the implausibility measure to determine, possibly through iterated tuning, whether a mismatch is due to parameter values or the parameterization itself, i.e., model discrepancy. Thus, history matching requires expert judgment in an essential manner. Note that while this aspect of expert judgment does not involve the use of GPs themselves, it is a necessary requirement for using GPs in history matching.

In sum, the above discussion speaks against the idea that by using ML models such as GPs in parameter tuning, this process becomes “objective” or “automatic”, such that the need for (different kinds of) expert knowledge is obsolete. This, of course, is not an argument against using these ML models in this context in principle.

### The progressives

Gentine et al. (2018) agree with Schneider et al. (2017a) that current convection parameterization schemes are responsible for biases that affect the predictive capabilities of GCMs. They propose to replace super-parameterizations (SPs, see section 2) with deep neural networks (DNNs) in the grid cells of GCMs to speed up computations. Gentine et al. write that ML models are suitable to overcome the convection parameterization deadlock because they “have been used in many applications where a clear physically based algorithm could not be defined” (p. 5743).

Gentine et al. use the so-called SPCAM3 (super-parameterized community atmosphere model), a GCM with SP, to generate simulated and labeled data, to train and test DNNs. The input variables include temperature, humidity, surface pressure and heat fluxes, while the output variables include temperature and humidity tendencies as well as heating tendencies. The first year of the simulation data was used for training, while the second year served as test data. The main results found by Gentine et al. are that predictions by DNNs show good qualitative agreement with those of SP. Quantitatively, DNNs and simulations agree well in midlevel regions and less well in boundary layers. Gentine et al. conclude that DNNs show good ability to reproduce some qualitative and quantitative aspects of the simulation data. The main advantage of the ML approach is that in preliminary tests, a GCM enhanced with DNNs is up to 10 times faster than the original SPCAM3. However, they also note several drawbacks: DNNs do not intrinsically conserve energy and moisture, which would be necessary for climate models in particular, and it is unclear how well the new model will generalize, i.e., how it will perform in situations not represented in the training data.

Rasp et al. (2018) continue Gentine et al.’s project. The main novelty is the integration of trained DNNs into the GCM. Rasp et al. add a further justification for replacing entire parameterizations with ML models: “To improve climate predictions, therefore, novel, objective, and computationally efficient approaches to subgrid parameterization development are urgently needed” (p. 9684). ML methods are supposed to be such a novel and ‘objective’ approach: “In this study, we explore whether deep learning can provide an objective, data-driven approach to using high-resolution modeling data for climate model parameterization” (p. 9684).

The experimental setup by Rasp et al. is similar to Gentine et al.; the investigation is based on SPCAM3, and the SPs are used to generate training data. The major difference, the integration of DNNs into the GCM to make projections, makes it possible to compare projections of the GCM enhanced with DNNs (called NNCAM) with projections of SPCAM3. There is an additional comparison between NNCAM and the same GCM with a traditional parameterization (called CTRLCAM). Rasp et al. report the following main results. NNCAM is 20 times faster than SPCAM3, and 8 times faster than CTRLCAM. NNCAM reproduced the mean tendencies of the benchmark simulation SPCAM. NNCAM is also able to reproduce qualitative aspects of climate variability, surpassing CTRLCAM, which exhibits well-known biases. Interestingly, NNCAM also approximately satisfies energy conservation, even though this was not explicitly enforced. Results concerning the generalization properties of NNCAM are mixed. If boundary conditions (sea surface temperature) are changed, the model is not able to reproduce projections for temperature changes of 4K, if it has not been trained with simulation data resulting from such changes. On the other hand, if the model has been trained with data from two extreme temperature scenarios, the model was able to reproduce intermediate scenarios. Thus, the model can interpolate, but it cannot extrapolate.

Rasp et al. explicitly address the argument of the conservative camp (Schneider et al. , 2017b) about the use of ML in the context of parameterizations. According to Rasp et al., the approach of the conservatives has several possible advantages, such as better generalization properties, reduction of the required amount of data, and the possibility of using components of the model for process studies. These advantages are grounded in the fact that known physical properties are hard-coded into the parameterizations. However, this approach has the problem of “heuristically finding the framework equations”. Note, again, the emphasis of the heuristic nature of formulating parameterizations.

Not all researchers in the progressive camp justify their approach through objectivity. Brenowitz and Bretherton (2018) trained DNNs on a near-global simulation model (CAM), and evaluated the predictions for convection in a single column. They did not couple the trained DNNs to a GCM, similar to Gentine et al. (2018). Brenowitz et al. provide a different motivation for the progressive approach: while ML methods could be used to tune parameters in traditional parameterizations, this approach is not ideal because the parameters of traditional parameterizations are designed to have a physical interpretation. If DNNs are used instead, this restriction is no longer necessary because their parameters are determined automatically.

Brenowitz and Bretherton (2019) continued their 2018 work; the authors coupled the DNNs to the dynamical core of a GCM, similar to Rasp et al. (2018). Brenowitz et al. stress that automatically tuning traditional parameterizations, which are “usually designed by human physical intuition, informed by high-resolution simulations and observations” (p. 2728) would be feasible, but caution that “these techniques can tune a few free parameters but may not scale to larger numbers of parameters. Moreover, existing parametrizations may not be flexible enough to be realistic in part because they have so few parameters.” (p. 2729).

In contrast to other progressives such as Rasp et al., Brenowitz et al. do not claim that utilizing ML models would lead to more objectivity. Rather, they point out that “ML models are typically trained by minimizing a loss function, such as the mean-square error (MSE) compared to some reference outputs from the training data; the choice of the loss function is subjective and a key to good performance” (p. 2729). Thus, similar to Schneider et al. (2017b), Brenowitz et al. emphasize that ML models themselves necessitate (subjective) choices.

The need for expertise when using ML methods in the context of parameterizations is further stressed in the discussion, where Brenowitz et al. write that at first, utilizing DNNs did not work as intended: “An attempt to couple a preliminary version of this NN to this GCM caused the model to blow up” (p. 2742). Brenowitz et al. then note that that adapting the DNNs as a parameterization replacement required human intervention: “The spatially extended simulations were stabilized by removing the upper atmospheric humidity and temperature from the NN inputs. This configuration can run stably indefinitely, without blowing up. Thus, stabilizing the parametrization required a rather crude human intervention. Future studies will need to explore automatic ways to discover true causal relationships and forestall model blow-up in a dynamically coupled setting” (p. 2742).

### Deep neural networks (ML for progressives)

The previous section provided examples of utilizing DNNs for replacing traditional parameterizations, while also showing that utilizing DNNs runs into several problems. First, DNNs sometimes show unphysical behavior, e.g., violation of conservation laws. Second, the integration of DNNs into GCMs yields problems of its own, in particular numerical instabilities. Third, DNNs are known to show poor generalization properties when used on out-of-distribution data. Morrison et al. (2020, Sec. 4.4). note two additional problems of utilizing ML models in the present context, which we add to the above list: Fourth, limited uncertainty quantification, and fifth a lack of interpretability of some ML models, DNNs in particular. What these problems show is that the use of DNNs to improve parameterizations poses novel challenges. We will now examine recent attempts to overcome some of these problems.

First, consider a proposal to tackle the problem of unphysical behavior. Beucler et al. (2019) note that traditional parameterizations exhibit empirical biases (“a lack of extreme precipitation events and unrealistic cloud structures”, p. 1); overcoming these is the main motivation for the authors to use DNNs. However, DNNs suffer from problems such as a lack of mass and energy conservation. Beucler et al. propose to overcome these problems by constraining DNNs to (approximately) satisfy conservation laws by i) using a suitable regularization term, which yields an approximate satisfaction of conservation laws and ii) by customizing the architecture of DNNs, such that they satisfy conservation laws exactly; see also (Beucler et al. 2021). Beucler et al. apply these methods to a climate model, SP-CAM with an aquaplanet boundary condition, similar to Gentine et al. (2018) and Rasp et al. (2018). Beucler et al. find that on both approaches, the generalization abilities of the ML models are significantly improved. This work is continued in Beucler et al. (2020), where the authors use physical rescaling to improve the generalization properties of DNNs. Thus, to solve the problem of unphysical behavior, it is useful to integrate physical constraints into ML models.

Second, consider a proposal to address numerical instabilities and a lack of interpretability. Brenowitz et al. (2020) investigate both SP-CAM, the climate model investigated by Gentine et al. (2018) and Rasp et al. (2018), and the coarse-grained global cloud resolving model investigated by Brenowitz and Bretherton (2018, 2019). Brenowitz et al. (2020) identify two problems. The first problem is that the coupling of DNNs replacing traditional parameterizations to GCMs leads to numerical instabilities (cf. Brenowitz and Bretherton 2019). This phenomenon occurs for both SP-CAM and the coarse-grained global cloud resolving model. The second, related problem is a lack of interpretability techniques to understand DNN parameterizations. Such interpretability techniques are necessary to understand the behavior of DNNs themselves, to overcome problems such as numerical instability; see Räz and Beisbart (2022) for a discussion regarding the importance of interpretability for scientific uses of DNNs. While techniques to interpret DNNs in general exist, these are unsuitable for the case of parameterizations. This necessitates the development of novel, domain-specific interpretability techniques.

Brenowitz et al. (2020) develop such domain-specific interpretability techniques, which allowed them to also develop new regularizations to overcome the numerical instabilities. In the summary, the authors write: “We hope that these interpretability techniques will aid in discovering more elegant solutions to the coupled stability problem and facilitate a more detailed exploration of neural network hyperparameters (e.g., depth) than has been possible in the past” (p. 4373). This means that these methods to better understand DNNs will help experts to improve machine learning parameterizations and, ultimately, the climate models. Expertise is necessary on at least two levels: To develop and improve domain-specific interpretability techniques, and to apply these techniques to improve parameterizations through ML models.

In sum, these examples show that the use of DNNs does not automatically lead to better predictions. Rather, utilizing DNNs in the context of parameterizations requires a different, novel kind of expert knowledge, viz. that of *adapting* DNNs to this particular domain. The expert knowledge is novel because it requires the integration of knowledge about DNNs and domain-specific knowledge.

## Discussion

With this conception of the two ML camps, conservatives and progressives, we return to the three kinds of subjectivity in expert judgment identified in Section 2.2 and discuss how the use of ML in parameterizations affects these aspects of subjectivity.

### Inductive skill / content knowledge

The design of the phenomenological laws that enter into the parameterization schemes require both inductive skills and content knowledge by the domain experts. This content knowledge is closely tied to the current state of a particular research field, in the present case, of the latest understanding of the climate phenomena which are to be represented in the parameterization. In the case studies we saw that a novel field, ML, is being utilized and applied to climate model parameterizations. This means that a novel kind of content knowledge is relevant, namely, that of ML and the structure these methods use to represent a given target. This expertise can in principle be provided by ML researchers, however, utilizing ML for parameterizations does not only necessitate expertise from this additional field. Rather, ML and climate model parameterizations have to be amalgamated because ML methods must be adapted to this novel context. Thus, this creates the need for novel expertise of a new subfield, “ML for climate modeling”.

While the amalgamation of fields is an issue that affects both conservatives and progressives, they are not affected equally. For the progressives, the inductive skill needed to replace entire parameterization schemes is greater, in that using ML instead of traditional parameterization schemes creates novel inductive problems. While parameterizations have well-known empirical biases, DNNs have several shortcomings: They show unphysical behavior (e.g., lack of energy and moisture conservation), and they are unreliable when used on out-of-distribution data. Both problems are particularly severe in the context of climate modeling (see Gentine et al. 2018 and Rasp et al. 2018). So it may be difficult for these new experts to answer the following key question: how are the relevant features of the target best or adequately captured within the framework of the new ML methods? Additionally, DNNs lack interpretability (Rudin 2019) and sometimes show a lack of numerical stability when coupled with climate models (Brenowitz et al. 2020). Climate modelers and ML experts are working on methods to overcome these difficulties of applying DNNs to replace parameterizations. However, doing this does not amount to automation or getting rid of the subjective elements linked to expert judgment. Rather, it requires both ML expertise, and mixed expertise to adapt DNNs to the context of parameterizations, e.g., to build DNNs that automatically satisfy conservation laws, or avoid numerical instabilities.

Overall, the degree of subjectivity that stems from the inductive skills and content knowledge of the expert does not decrease if ML methods are introduced. Rather, the use of ML models creates different and novel challenges for experts in terms of amalgamating different fields and applying theoretical knowledge in a novel context, all of which continue to involve some subjective features of the expert.

### Practical experience / track record

The design/use of parameterizations is a matter of practical experience and know-how, which involves tacit knowledge that is difficult to be made explicit and can only be acquired through an individual’s experience. As previously discussed, the way in which this aspect of expertise is evaluated is via the track record or the repeated success of the expert using the practical knowledge in question. When we apply this concept to the use of ML in parameterizations we find a problem, since gaining practical experience and building a track record requires some time, but the practical experience for utilizing ML methods in this way has to be acquired anew and thus requires some time before being ‘operational’. This situation is especially true for the progressive group, who are calling for the complete replacement of the parameterization with new ML methods like DNNs. Given this, we argue that one should actually expect that the degree of subjectivity would increase through the use of ML methods rather than decrease, as currently there is little robust track record for any expert. This however, is due to the simple fact that the use of these methods is relatively new, and there has not been enough time for practical experience and further expertise to be acquired and implemented. Thus, this may only be a temporary problem and could diminish eventually, but as it currently stands one cannot guarantee a robust track record will be produced in the short term for the use of DNNs or ML methods more generally in this context.

### Values / bias

Expert judgments can be biased by making choices in parameterizations based on a preference for certain purposes, for example, making good predictions for a certain geographic region, thereby (possibly) neglecting other regions. Such biases need not be due to modeling choices, but can also be due to the availability of climate data for some regions such as the Global North, and a lack of data for the Global South. As previously discussed, these biases affect the practical application of the parameterization as they are potentially only epistemically justified for use within the concerned region (or for a given climate variable). Additionally, depending on the application of the parameterization within the larger model, there can be additional concerns regarding the reliability of the outputs, if incompatible values/biases were used to construct other aspects of the model. From this, we see that the use of values in traditional parameterizations only comes into play when applied to real world circumstances and can only be evaluated or managed on a case by case basis.

In the cases of using ML models in parameterizations, this kind of subjectivity does not appear to play an important role. This, however, is probably due to the fact that these studies focus on the development of novel methods. Some works (such as Gentine et al. 2018) characterize their contribution as proof-of-concept, i.e., as establishing the viability of methods in principle, not necessarily utilizing these methods in practical circumstances. Also, many of the above contributions rely on synthetic/simulation data and use idealized scenarios such as aquaplanets. This means that issues related to data availability and the focus on certain geographic regions are not considered—for the time being.

However, we should expect that these issues resurface eventually, in particular once ML-parameterizations are used on real data. In the conservative camp, biases due to the use of traditional parameterization schemes will persist. The challenge for the progressive camp will be to adapt the ML tuning process to different purposes. Methods such as DNN are only reliable to the extent that data relevant for a particular purpose is available. This raises the above mentioned worry that for regions where data is scarce, prediction quality and epistemic justification will be weaker than for regions with abundant data. This is very much analogous to known cases of biases of DNNs; see, e.g., Rezk et al. (2022) on the consequences of the underrepresentation of dark skin tones for cancer detection by DNNs. Unequal representation in the data will lead to a differential in prediction quality and epistemic warrant, and thus, ultimately, to an inadequacy for certain purposes.

## Conclusion

Parameterization and parameter tuning are central aspects of climate modeling, and there is widespread consensus that these procedures involve certain (possibly irreducible) subjective (“artisanal”) elements, the nature of which we have discussed in Sect. 2. Even if these subjective elements are not necessarily epistemically problematic, there is a general intuitive appeal—at least in parts of the climate science community—for replacing them with more objective (automated) methods. In this sense, machine learning techniques are very natural candidates. Machine learning has been applied in different ways to climate model parameterization in the recent literature, and we have distinguished the conservatives from the progressives according to their willingness to abandon standard parameterization and tuning procedures for machine learning methods (Sect. 3). Relying on several case studies, we have argued that the subjective elements we identified in the context of standard parameterization and tuning procedures are still present—albeit in novel forms—when these standard procedures are (partly or fully) replaced by machine learning methods (Sect. 4). The case studies show that using ML in this context poses various new challenges: Building the expertise required to amalgamate ML and parameterizations will take time, and efforts to build physical constraints into ML models, and to develop domain-specific interpretability techniques, have just started. This does not affect the relevance of machine learning methods for climate model parameterization and tuning (in particular when it comes to the computational cost), but it does call for a careful epistemic attitude, especially with respect to the (possible lack of) representativity and the potential biases of the training data sets.

## Data Availability

Not applicable as no data was used in this research.

## Footnotes

1 The global climate models of the Coupled Model Intercomparison Project, currently in phase 6 (CMIP6), which underlies the climate projections made in the latest assessment report AR6 of the IPCC (2021) have an atmospheric horizontal resolution between 50 and 250 km roughly (see IPCC 2021, ch. 1, fig. 1.19).

2 We take experts in this context to be climate modelers, those who partake in the building and implementation of climate models.

3 In this sense, as noted in Hourdin et al. (2017), climate model tuning is in many ways akin to parameter estimation in statistics and is similar to what is referred to as ‘calibration’ in other domains of numerical modeling (for a critical discussion, see Touzé-Peiffer 2021).

4 With testimonial knowledge generally characterized as belief that is grounded in a written or spoken statement provided by another individual (Lackey 2010).

5 For an overview of the required or desirable expert traits, see Martini (2014, 2015, 2020) and the references within.

6 In a recent review of ‘physics-informed’ applications of machine learning in weather and climate modeling, (Kashinath et al. , 2021) suggest that machine learning “offers novel approaches to replacing approximations or empirical parameters with data-driven learned counterparts” (p. 6) and more specifically that machine learning “could be employed to find the optimal set of critical parameters in weather and climate models” (p. 7).

7 Convection and cloud-related parameterization is the focus of particular attention in the climate science community because it is considered to be the main source of uncertainty in climate change projections (Bony et al. 2015, Schneider et al. 2017a).

8 Cloud physics can be represented using even higher resolution models, such as large-eddy simulations, which have a horizontal resolution of a few hundreds meters.

9 We thank an anonymous reviewer of this journal for pointing out this argument.

## References

[CR1] Beisbart C, Räz T (2022) Philosophy of science at sea: clarifying the interpretability of machine learning. Philosophy Compass, e12830

[CR2] Beucler T, Pritchard M, Gentine P, Rasp S (2020) Towards physically-consistent, data-driven models of convection. In IGARSS 2020-2020 IEEE international geoscience and remote sensing symposium. IEEE, pp 3987–3990

[CR3] Beucler T, Pritchard M, Rasp S, Ott J, Baldi P, Gentine P (2021). Enforcing analytic constraints in neural networks emulating physical systems. Phys Rev Lett.

[CR4] Beucler T, Rasp S, Pritchard M, Gentine P (2019) Achieving conservation of energy in neural network emulators for climate modeling. arXiv:http://arxiv.org/abs/1906.066221906.06622

[CR5] Bony (2015). Clouds, circulation and climate sensitivity. Nature Geosc.

[CR6] Brenowitz ND, Beucler T, Pritchard M, Bretherton CS (2020). Interpreting and stabilizing machine-learning parametrizations of convection. J Atmospher Sci.

[CR7] Brenowitz ND, Bretherton CS (2018). Prognostic validation of a neural network unified physics parameterization. Geophys Res Lett.

[CR8] Brenowitz ND, Bretherton CS (2019). Spatially extended tests of a neural network parametrization trained by coarse-graining. J Adv Model Earth Syst.

[CR9] Couvreux et al (2020) Process-based climate model development harnessing machine learning: I. a calibration tool for parameterization improvement. J Avances Model Earth Syst 13:e2020MS002217

[CR10] Douven I (Forthcoming) Explaining the success of induction. British J Philo Sci. 10.1086/714796

[CR11] Edwards P (2010) A vast machine: computer models, climate data, and the politics of global warming. MIT Press, Cambridge, MA

[CR12] Gentine et al (2018) Could machine learning break the convection parameterization deadlock? Geophys Res Lett 45:5742–5751

[CR13] Guillemot H (2017) How to develop climate models ? the “gamble” of improving parameterization. In Heymann, M., Gramelsberger, G., and Mahony, M., editors, Culture of prediction in Atmospheric and Climate Science. Epistemic and cultural shifts in computer-based modelling and simulation, pp 120–136. Routledge

[CR14] Hourdin et al (2017) The art and science of climate model tunig. Bullet Amer Meteorological Soc 98(589–602)

[CR15] Intemann K (2015). Distinguishing between legitimate and illegitimate values in climate modeling. Eur J Philo Sci.

[CR16] IPCC (2021) Climate change 2021: the physical science basis. Contribution of working group I to the sixth assessment report of the intergovernmental panel on climate change. Intergovernmental panel on climate change

[CR17] Jebeile J, Lam V, Räz T (2021). Understanding climate change with statistical downscaling and machine learning. Synthese.

[CR18] Kashinath et al (2021) Physics-informed machine learning: case studies for weather and climate modelling. Philo Trans Royal Soc 79:20200093 10.1098/rsta.2020.0093 33583262

[CR19] Katzav J (2013). Hybrid models, climate models, and inference to the best explanation. Brit J Phil Sci.

[CR20] Kawamleh S (2021). Can machines learn how clouds work?: The epistemic implications of machine learning methods in climate science. Philo Sci.

[CR21] Knüsel B, Baumberger C (2020). Understanding climate phenomena with data-driven models. Stud Hist Phil Sci.

[CR22] Lackey J (2010). Routledge companion to epistemology, chapter testimonial knowledge.

[CR23] Lenhard J, Winsberg E (2010). Holism, entrenchment, and the future of climate model pluralism. Stud Hist Philos Modern Phys.

[CR24] Lloyd EA (2015). Model robustness as a confirmatory virtue: the case of climate science. Stud Hist Philos Sci Par.

[CR25] Martini C (2014). Experts in science: a view from the trenches. Synthese.

[CR26] Martini C (2015). Expertise and institutional design in economic committees. J Econ Method.

[CR27] Martini C (2020) The epistemology of expertise, chapter 12. Routledge, 1st edn

[CR28] Mauritsen et al (2012) Tuning the climate of a global model. J Avances Model Earth Syst 4:M00A01

[CR29] McNeall D, Williams J, Betts R, Booth B, Challenor P, Good P, Wiltshire A (2020). Correcting a bias in a climate model with an augmented emulator. Geosci Model Dev.

[CR30] Morrison H, van Lier-Walqui M, Fridlind AM, Grabowski WW, Harrington JY, Hoose C(2020) Confronting the challenge of modeling cloud and precipitation microphysics. J Adv Model Earth Syst 12 10.1029/2019MS001689 PMC7507216 32999700

[CR31] O’Gorman PA, Dwyer J (2018). Using machine learning to parameterize moist convection: potential for modeling of climate, climate change, and extreme events. J Avances Model Earth Syst.

[CR32] Parker WS (2020). Model evaluation: an adequacy-for-purpose view. Philos Sci.

[CR33] Proske U, Ferrachat S, Neubauer D, Staab M, Lohmann U (2021) Assessing the potential for simplification in global climate model cloud microphysics. Atmospher Chem Phys Discussions, pp 1–40

[CR34] Randall D, Khairoutdinov M, Arakawa A, Grabowski W (2003). Breaking the cloud parameterization deadlock. Bullet Amer Meteorological Soc.

[CR35] Rasp S, Pritchard MS, Gentine P (2018). Deep learning to represent subgrid processes in climate models. Proc National Acad Sci.

[CR36] Räz T, Beisbart C (2022) The importance of understanding deep learning. Erkenntnis 10.1007/s10670-022-00605-y PMC11090801 38751773

[CR37] Reichstein M, Camps-Valls G, Stevens B, Jung M, Denzler J, Carvalhais N (2019). Deep learning and process understanding for data-driven Earth system science. Nature.

[CR38] Rezk E, Eltorki M, El-Dakhakhni W (2022). Improving skin color diversity in cancer detection: deep learning approach. JMIR Dermatol.

[CR39] Rio C, Del Genio AD, Hourdin F (2019). Ongoing breakthroughs in convective parameterization. Current Climate Change Reports.

[CR40] Rudin C (2019). Stop explaining black box machine learning models for high stakes decisions and use interpretable models instead. Nature Mach Intell.

[CR41] Schmidt GA, Bader D, Donner LJ, Elsaesser GS, Golaz J-C, Hannay C, Molod A, Neale RB, Saha S (2017). Practice and philosophy of climate model tuning across six us modeling centers. Geosci Model Dev.

[CR42] Schneider et al (2017a) Climate goals and computing the future of clouds. Nature Climate Change 7:3–5

[CR43] Schneider et al (2017b) Earth systemmodeling 2.0: A blueprint for models that learn from observations and targeted high-resolution simulations. Geophys Res Lett 44:12396–12417

[CR44] Stevens B, Bony S (2013). What are climate models missing?. Science.

[CR45] Touzé-Peiffer L (2021) Paramétrisation de la convection atmosphériquue dans les modèles numériques de climat – Pratiques et enjeux épistémologiques. PhD thesis, Sorbonne-Université, spécialité doctorale “Sciences de l’Environnement”

[CR46] Williams CK, Rasmussen CE (2006). Gaussian processes for machine learning.

[CR47] Williamson D, Goldstein M, Allison L, Blaker A, Challenor P, Jackson L, Yamazaki K (2013). History matching for exploring and reducing climate model parameter space using observations and a large perturbed physics ensemble. Climate Dynamics.

[CR48] Williamson DB, Blaker AT, Sinha B (2017). Tuning without over-tuning: parametric uncertainty quantification for the nemo ocean model. Geosci Model Dev.

[CR49] Winsberg E (2018). Philosophy and climate science.

